# Application of Graphene Nanoplatelets in Supercapacitor Devices: A Review of Recent Developments

**DOI:** 10.3390/nano12203600

**Published:** 2022-10-13

**Authors:** Eleri Anne Worsley, Serena Margadonna, Paolo Bertoncello

**Affiliations:** Future Manufacturing Research Institute, Faculty of Science and Engineering, Swansea University Bay Campus, Fabian Way, Crymlyn Burrows, Swansea SA1 8EN, UK

**Keywords:** graphene nanoplatelets, supercapacitor, electric double-layer, energy storage devices

## Abstract

As worldwide energy consumption continues to increase, so too does the demand for improved energy storage technologies. Supercapacitors are energy storage devices that are receiving considerable interest due to their appealing features such as high power densities and much longer cycle lives than batteries. As such, supercapacitors fill the gaps between conventional capacitors and batteries, which are characterised by high power density and high energy density, respectively. Carbon nanomaterials, such as graphene nanoplatelets, are being widely explored as supercapacitor electrode materials due to their high surface area, low toxicity, and ability to tune properties for the desired application. In this review, we first briefly introduce the theoretical background and basic working principles of supercapacitors and then discuss the effects of electrode material selection and structure of carbon nanomaterials on the performances of supercapacitors. Finally, we highlight the recent advances of graphene nanoplatelets and how chemical functionalisation can affect and improve their supercapacitor performance.

## 1. Introduction

Predictions suggest that global energy supply must double by 2050 to keep pace with the ever-increasing demand [1], leading to a potential energy crisis. Such rapidly increasing energy demand is subsequently causing a growing need for more effective energy storage technologies, which will require better power densities to aid faster energy storage and release, alongside better energy densities for storing larger amounts of energy. However, there is currently no simple one-size-fits-all solution to this issue; the best storage solution for any application depends strongly on its specific requirements and properties. To assess the most suitable solution, many specifications must be considered including lifetime, reliability, storage capacity, cost and environmental impact [2].

**Figure 1:**
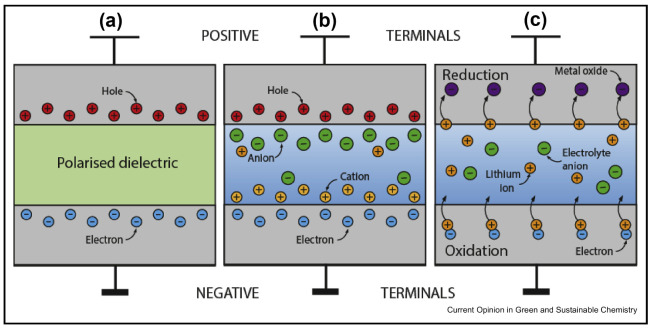
Schematic illustration of the differences in electrical charge storage mechanisms between (**a**) a dielectric capacitor with wide charge separation and polarised dielectric, (**b**) an EDLC with much closer charge separation partially provided by ions in the electrolyte, and (**c**) a Li ion-type redox battery with charge transfer occurring across the electrode-electrolyte interface. The thin upper line on each represents the positive terminal, while the thick lower line is the negative. Reprinted with permission from Ref. [5]. 2019, Elsevier.

Electrical energy can be stored in two main ways; directly or indirectly, with both utilised in current storage technologies including batteries, fuel cells and supercapacitors. Indirect storage, such as conventional batteries, stores the electrical energy as chemical potential energy. The energy is discharged when the reagents undergo Faradaic redox reactions and release electric charges that flow between two electrodes of differing potential [3]. This causes electron transfer to occur across the surface of the electrodes, leading to a change in the oxidation state or chemical composition of the electrode material. This mechanism is sometimes also referred to as Faradaic electrical energy storage. In direct storage, also known as non-Faradaic electrical energy storage, charges are stored electrostatically on the negative and positive electrodes. This is the storage mechanism employed by the most common supercapacitor technology, electric double-layer capacitors (EDLCs). Both mechanisms involve reversible processes in which chemical potential energy is converted directly into Gibbs free energy. However, while non-Faradaic systems have a theoretically infinite reversibility, Faradaic systems possess only a limited reversibility. This issue arises as a result of the chemical interconversions of the electrode materials, which often include irreversible phase changes that limit the device lifetime to only hundreds or thousands of cycles [4]. Capacitors and supercapacitors ideally undergo no chemical or phase transitions, since charging and discharging requires only excess and deficiency of electrons at the relevant electrode plates [3]. Unfortunately, they do not operate as purely EDL devices in practice, and always have a small yet noteworthy pseudocapacitive contribution at an estimated 1–5% of their capacitance [4]. This is believed to be due to the Faradaic reactivity of surface functionalities, which depend on the origin and pre-treatment conditions of the carbon. In comparison, pseudocapacitor and redox devices, including batteries, exhibit up to an estimated 10% of their capacitance as EDL capacitance [3]. Hence, supercapacitors still experience a limited cycle life, albeit orders of magnitude higher than that of batteries. A simple schematic illustrating the difference between indirect storage in Li-ion batteries and direct storage in EDLCs can be seen in Figure 1, with a further comparison to a dielectric capacitor also illustrated [4].

**Figure 2:**
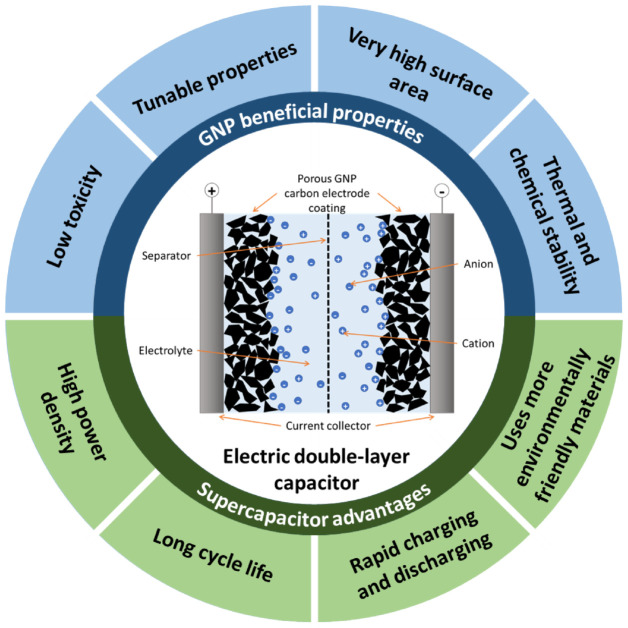
Overview diagram of the content of the paper, including a simple diagram of an electric double-layer capacitor, the beneficial properties of graphene nanoplatelets for supercapacitors, and the advantages for supercapacitors as an energy storage technology.

This review will focus on supercapacitors, which are a relatively new reversible electrochemical energy storage technology in comparison to batteries and fuel cells. The main two classifications of supercapacitor are defined by their storage mechanism: electric double-layer capacitance (EDLC) is associated with charging and discharging of the electric double-layer (EDL) at the electrode-electrolyte interface, whereas pseudocapacitance is caused by redox reactions and electrosorption processes [3]. More recent advances have also studied asymmetric and hybrid supercapacitors, which combine the properties of EDLC and redox reactions [3,6,7]. At its most simple, a supercapacitor consists of two electrodes separated with an electrolyte and semi-permeable membrane, known as a separator, which allows charges to travel through the electrolyte between both electrodes while keeping the electrodes from touching each other [6]. EDLC devices and electrodes will be the focus of this paper, with some pseudocapacitance introduced through additional functionalisation. A schematic diagram of a basic EDLC device is shown in Figure 2, showing all the basic components.

**Figure 3:**
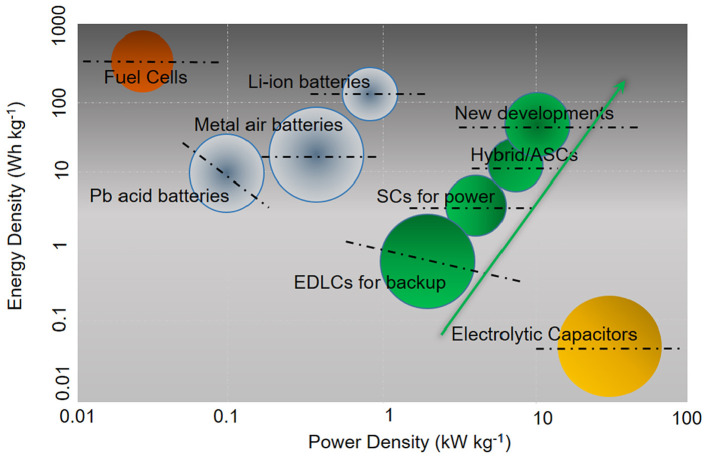
Illustration of Ragone Plot for performance comparison of various energy storage and conversion systems displaying the progress of energy and power density of the different types of supercapacitors and which position future devices are expected to obtain. Reprinted with permission from Ref. [7]. 2020, Elsevier.

The properties of EDLC supercapacitors can be considered as lying partway between a conventional dielectric capacitor and a traditional battery. However, they possess a higher energy density than dielectric capacitors and a higher power density than LIBs, as illustrated in Figure 3 [7,8]. The first practical application of EDL capacitance was proposed in a patent granted to Becker in 1957 [9]. The patent detailed a “Low Voltage Electrolytic Capacitor” which stored energy by holding charges in a double layer at the interface between a porous carbon material and an aqueous electrolyte. Though the basic principles are still the same, supercapacitor design and performance has since been developed and improved considerably, with many different device types now available [3,7]. Currently, due to their high power density, supercapacitors are being utilised in many high-power areas including hoisting machinery, wind power generation and hybrid electric vehicles and racing cars, since the high power output allows for acceleration on a very short timescale [10]. In addition to the benefits for fast acceleration in electric vehicles, the high capacitance provides advantages for regenerative braking; supercapacitors are able to recapture kinetic energy through regenerative braking at least 20% more efficiently than batteries [4,11]. However, a goal for future energy storage technology developments is to succeed in providing both high energy density and high power density simultaneously in a single system [2].

The most widely used supercapacitor electrode materials, particularly for EDLC devices, are high surface area carbon-based nanomaterials [2]. This is due to the many beneficial properties that this class of materials possess, from porous structure to corrosion resistance [12]. The carbon electrode material under analysis in this review will be graphene nanoplatelets, and the rationale behind the electrode material selection will be discussed in Section 2.3. A summary of the beneficial properties of GNPs that make them a suitable choice for a supercapacitor electrode material is described in Figure 2.

Within this review, the fundamentals of supercapacitor functionality will be discussed first and a brief description of pseudocapacitance will be given, before focusing on the EDL mechanism and EDLC devices. The problems pertaining to current supercapacitor technology will then be explored, alongside how such issues can be avoided through the choice of carbon nanomaterial and intelligent design of the porous structure. Current all-carbon based technologies being developed to overcome the drawbacks of traditional supercapacitor devices will be examined. Finally, the benefits and current research surrounding various functionalised GNP electrode materials will be reviewed. There are undoubtedly many facets to supercapacitor design beyond the electrode materials, from the binder and solvent used in the electrode ink [13,14,15,16,17,18,19] to the electrolyte choice [20,21,22,23,24,25,26], which can both have a considerable effect on the electrochemical performance of the device. Furthermore, many of the electrode materials to be discussed could also be candidates for battery technologies [27,28,29,30,31,32,33,34,35]. However, those topics are both beyond the scope of this review.

## 2. Background on Supercapacitor Technologies

### 2.1. Electric Double-Layer Capacitance

A standard symmetrical EDLC device uses two identical activated carbon electrodes and relies on the electric double layer phenomenon to store the charges [8]. On application of a potential difference, δE, across the supercapacitor electrodes, electrons are transported from one electrode to the other through the external circuit containing a polarising device, which sets up a charge difference between the two plates. The polarising device can be a power supply, a battery or a regenerative breaking dynamo in an electric vehicle [3]. These positive and negative charge densities on the supercapacitor plates are then matched by the accumulation of net equal and oppositely charged ions from the electrolyte on the metal surface.

The dependence on the electrode potential can be seen in the calculation of the double-layer capacitance:

(1) $$C_{dl}=q/E=\delta q/\delta E=dq/dE,$$

in which *E* is the electrode potential and *q* is the charge on the electrode [3].

**Figure 4:**
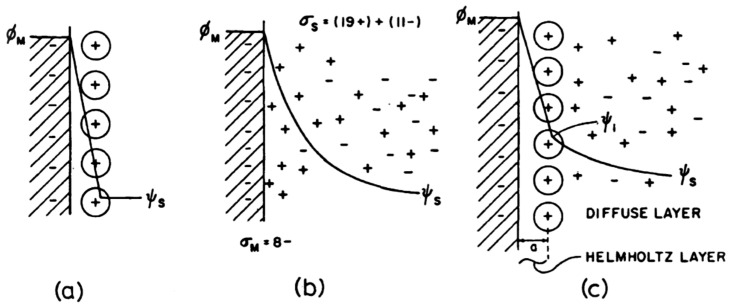
The changing model of the double layer: (**a**) Helmholtz model, (**b**) Gouy point-charge model, (**c**) Stern model for finite ion size with thermal distribution, combining the Helmholtz and Gouy models. Reprinted with permission from Ref. [3]. 1999, Springer.

The electric double layer that exists at the boundary between an electrode and electrolyte is a phenomenon that is now well established. However, the exact mechanisms and structure of this double layer has been a topic of long debate, with the models evolving over many decades. Regardless of the specific mechanism, it is agreed that the double layer controls the adsorption properties, affects charge transfer reaction rates, and is the place where energy storage occurs in an EDLC device [3]. The initial concept of a double layer was modelled by von Helmholtz in 1853 to illustrate their perception of the distribution of opposite charges at the interface of colloidal particles [8,36]. This model is now referred to as the Helmholtz double-layer model, and is illustrated in Figure 4a [3]. An altered model, proposed by Gouy In 1910, realised that due to thermal fluctuations governed by the Boltzmann principle, the ions would not inhabit a compact, static layer. Instead, the model described the ions as a diffuse distribution of positive and negative point charges, as illustrated in Figure 4b [3]. However, Gouy’s assumption of the ions as point charges lead to two major failures in the model: an incorrect potential profile and local field near the electrode surface and hence an overestimation of the double-layer capacitance [3].

**Figure 5:**
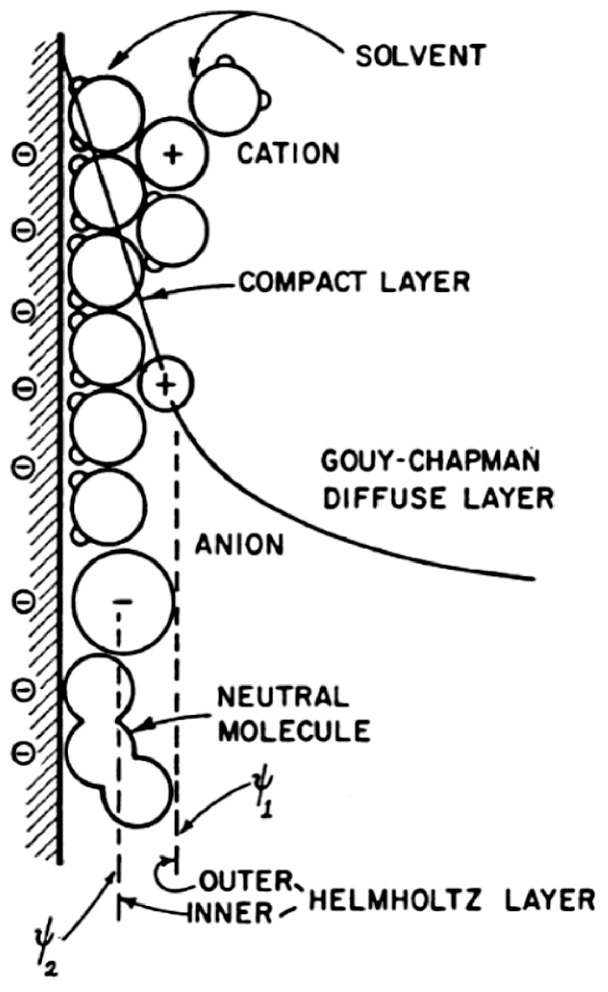
The Grahame model of the double layer, showing different regions for adsorption of smaller, more-hydrated cations and larger, less-hydrated anions. Reprinted with permission from Ref. [3]. 1999, Springer.

A modified model proposed by Stern in 1924 overcame these overestimation problems by combining ideas from both the Helmholtz and Gouy models. It was identified that the inner region of the ion distribution could be treated as a compact region of adsorption, like in the Helmholtz model, while the region beyond this initial layer could be regarded as a diffuse distribution of charges, as in the Gouy model [3]. This is depicted in Figure 4c. The geometrical limit of this compact layer could be readily defined by taking into account the finite size of the ions. Furthermore, the introduction of finite-sized ions automatically avoided the overestimation of capacitance from the Gouy model. This led to a model which contains both a Helmholtz-type compact double layer ($C_{H}$) and a diffuse Gouy-type region ($C_{diff}$). A final model was developed by Grahame in 1947, which added a distinction between the inner and outer Helmholtz layers, corresponding to the differing distances of closest approach experienced by cations and anions at the electrode surface [3]. Therefore, there are three distinct regions present in the Grahame model: the inner Helmholtz layer, the outer Helmholtz layer, and the diffuse ion distribution region, as shown in Figure 5. Since the distance of closest approach tends to be smaller for anions than cations, the inner layer capacitance of the positive electrode is usually up to twice as high as that of the corresponding negative electrode, though this does depend on the choice of metal, electrolyte, and solvent [3].

**Figure 6:**
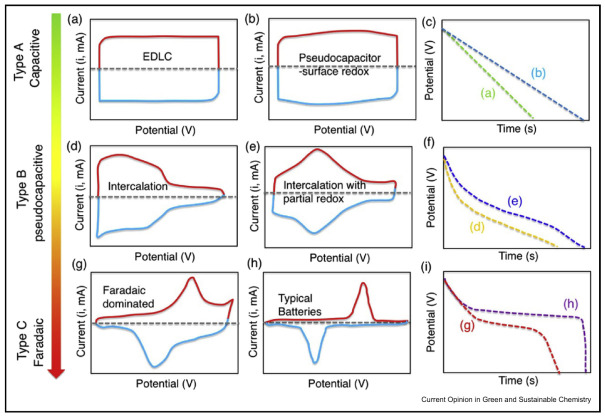
Schematic CV traces for different energy storage materials and their respective galvanostatic discharge curves. Each galvanostatic discharge curve is labelled with the letter of the corresponding CV trace. (**a**–**c**) Type A materials are distinctly capacitive, which includes EDLCs; (**d**–**f**) Type B are pseudocapacitive materials which are diffusion and/or intercalation limited; (**g**–**i**) Type C materials show a Faradaic battery-like response. Reprinted with permission from Ref. [4]. 2019, Elsevier.

The EDL capacitance of an electrode in an EDLC can be calculated using:

(2) $$C=\frac{\epsilon_{r}\epsilon_{0}}{d}A,$$

where $\epsilon_{r}$ and $\epsilon_{0}$ are relative permittivity of the electrolyte and vacuum permittivity, respectively, *A* is the effective contact area between the electrode and electrolyte and *d* is the thickness of the EDL [2,37]. As mentioned above, electrical energy storage devices are characterised using their electrochemical performance; most notably their energy and power density. In an ideal case with constant capacitance, a linear charge-discharge signature and a rectangular CV curve is achieved, as in Figure 6a–c. When this is true, the energy density *W* and the maximum power density $P_{m}ax$ can be expressed as

(3) $$W=\frac{1}{2}CV^{2},$$

(4) $$P_{max}=\frac{V^{2}}{4R},$$

where *C* is the capacitance, *V* is the operating voltage, and *R* is the equivalent series resistance of the device [11]. In a more general sense, the discharged energy and the power can be calculated using

(5) W=∫V(t)I(t)dt,

(6) P=V(t)I(t),

where *V* is the potential and *I* is the current during discharge time *t* [11]. As can be seen in Figure 6, different types of electrical energy storage have very distinct CV curves and charge-discharge traces, which can be used to identify how closely a device aligns to the ideal properties. A purely EDLC device shows a characteristic rectangular CV trace, in comparison to the distinct redox related peaks seen for pseudocapacitors and batteries [4].

Commercial EDLC devices usually have energy densities in the range 3–10 Wh Kg${}^{-1}$ [2], while LIBs can reach 180 Wh Kg${}^{-1}$ [38]. The significance of power and energy densities for electrical energy storage can be illustrated by considering the situation if a storage device was used in an electric vehicle; the power density would dictate how fast the vehicle could go, whereas the energy density would govern the distance that could be travelled on a single charge [39]. Furthermore, as mentioned above, they have considerably longer cycle lives than batteries and are able to be fully charged or discharged in a matter of seconds to minutes due to their high power density [2,4]. They also experience relatively low voltage and current hysteresis between charging and discharging processes [2,8].

### 2.2. Pseudocapacitance

Pseudocapacitance is a phenomenon that arises in some electrosorption and Faradaic redox processes and at electrode surfaces of oxide films, such as $RuO_{2}$, $IrO_{2}$, and $Co_{3}O_{4}$, where there is a continuous dependence of charges *q* on the electrode potential *V* [3]. In contrast to double-layer capacitance, pseudocapacitance is Faradaic in nature, meaning charges pass across the double layer in the same manner as with battery charging and discharging. It also involves chemical changes of state of the reactant in order to facilitate electron transfer [3]. As a result of the constraints of thermodynamics, a special relation arises between the degree of charge acceptance (δq) and the potential change (δV) that results in the emergence of the derivative dq/dV. This quantity is equivalent to a capacitance and can be experimentally measured as such. However, it is referred to as a *pseudo*-capacitance since it emerges in such a different manner to classical electrostatic capacitance [3]. Pseudocapacitors can operate at much higher specific capacitances than EDLCs since the redox reactions allow charges to be stored not only at the surface, but also in the bulk of the material [7]. However, they suffer from poor cycling stability, poor electrical conductivity and low durability when charging and discharging [2,8] in addition to lower power density due to the slower reaction kinetics [7]. Thus, constructing supercapacitor electrodes from solely pseudocapacitive materials would increase the overall capacitance, but at the expense of the power density and cycle life [8].One method to overcome the limitations of individual EDLC and pseudocapacitive materials is to combine the two into composites with a conductive carbon backbone supporting high capacitance pseudocapacitive components [8]. These improvements through structural modification and functionalisation will be discussed in more detail in Section 3.2.1.

### 2.3. Electrode Material Selection

Carbon-based materials are one of the most commonly used supercapacitor electrode materials due to their many desirable properties, including a designable porous structure, high surface area, good electric conductivity, corrosion resistance, and stability in a range of electrolytes, temperatures, and potentials [8,10]. Carbon materials for EDLC electrodes must have a high specific surface area (SSA), on the order of 1000 m${}^{2}$g${}^{-1}$, good inter- and intra-particle conductivity in porous matrices and good electrolyte accessibility to the internal pore surface area. The material should also be designed to have a good specific capacitance and minimal self-discharge rates, and so should be free of impurities [3]. Suitability can be improved through activation of the carbon, which creates a highly porous structure with a very high surface area, thus increasing the specific capacitance [8]. Activation can be used to generate porous carbon nanomaterials from pre-existing carbon sources such as coal, coconut shells or wood. The carbon-containing precursor material generally undergoes thermal carbonization, before receiving a physical or chemical activation pre-treatment [3]. The specific properties of the final carbon material, such as the SSA and pore properties, depend on both the precursor carbon material and the chosen activation process [40]. Most commercial EDLCs use ultrapure steam-activated carbon derived from coconut shells, while chemical activation is mainly only used at a laboratory scale [11].

Activated carbons (ACs) are a widely used electrode material in commercial applications due to their wide availability, low cost and relative ease of large-scale manufacture. ACs have a broad range of pore sizes and a high SSA, usually ranging from 1000 to 2000 m${}^{2}$g${}^{-1}$ and have been shown to have a long cycle life of >10${}^{6}$ cycles and a specific capacitance of 100 to 300 F g${}^{-1}$ when utilised in supercapacitor devices with non-aqueous electrolytes [40]. Carbon nanotubes (CNTs) have been investigated for supercapacitor electrodes in lab-scale trials and have demonstrated moderate specific capacitance of up to 100 F g${}^{-1}$ [40,41]. CNTs generally have quite high SSA of 100 to 1000 m${}^{2}$g${}^{-1}$, however, the internal surface area is not usually accessible to the electrolyte due to ion diffusion limitations. Therefore, only the external surfaces of the CNTs contribute to the specific capacitance [40]. This, alongside the high cost and difficulty of manufacture, make CNTs a less appropriate supercapacitor electrode material currently.

Since its discovery in 2004 [42], graphene has dominated many fields of materials and technology research due to its wide array of properties, in particular its extremely high surface area, excellent electric conductivity and high electron mobility [2,8]. A single sheet of graphene has a very high theoretical SSA of 2630 m${}^{2}$g${}^{-1}$ and a theoretical specific capacitance of 550 F g${}^{-1}$[39,40]. Graphene and graphene-based materials have become a popular choice for electrode materials due to their high power capability [43,44,45,46]. However, commercial powders usually suffer from undesirable properties, such as partial restacking and agglomeration due to the π-π interaction of graphene sheets, which greatly reduces the effective surface area and lowers the electrochemical performance [8,11,37]. This causes the measured specific capacitance of graphene-based materials to be much lower than the theoretical values. The use of graphene in commercial-scale electrode production is also inhibited greatly by the high production costs currently associated with graphene [40].

Due to these prohibitive properties of graphene, many researchers are turning to other graphene-based or graphene-like materials, which share some desirable properties while improving upon the negative attributes. Three-dimensional aerogels are one material type that is currently being explored as a graphene replacement in supercapacitor electrodes [47,48,49]. Graphene aerogels are ultra-lightweight with a high specific surface area, and encompass a high volume of pores which allow easy soaking by the electrolyte alongside fast charge-discharge processes [47]. The three-dimensional architecture of the graphene aerogel aids in the prevention of graphene restacking and allows the high surface area to be maintained. Another material which has recently come under investigation for supercapacitor electrode materials is graphene nanoplatelets (GNPs) [50,51,52,53,54,55,56,57,58,59], also known as few-layer graphene (FLG). GNPs have many beneficial properties with regard to supercapacitor design, including low toxicity, very high surface area, thermal and chemical stability in a range of conditions and a hierarchical porous structure [55,56]. These properties can be tuned to the requirements of the desired application through alteration of the pore sizes, graphene sheet spacing, production methods and activation [53,57]. The recent innovations in GNP supercapacitor electrodes will be described in detail in this review.

### 2.4. Drawbacks of Conventional Supercapacitors

Current supercapacitor research is mainly focused on improving upon a number of key challenges present in current device manufacturing [3]:

1. Low energy density,
2. Limited specific capacitance and operating voltage,
3. Poor electrolyte accessibility,
4. Porous structure optimisation,
5. Agglomeration of carbon nanomaterials,
6. Difficulty with industrial scale production.

It is difficult to balance the desired properties of the porous electrodes with overcoming these problems, as they are often interlinked and contradictory. For example, the porous structure is important as accessible pores are needed for electrolyte pathways to maximise conductivity, alongside a highly accessible surface area in order to reach the highest possible specific capacitance [3]. However, the porous structure that best increases the electrolyte accessibility is not necessarily the same one which has the highest surface area. This will be examined further in Section 2.5.

The electrochemical performance of both aqueous and non-aqueous EDLCs is limited by the specific capacitance of the device, which in turn is attributed to the inherent properties of the original carbon materials [2,21]. Finite conductivity, narrow operating voltage and the aforementioned incomplete utilisation of the active area all affect the specific capacitance of EDLCs and cause it to be generally limited to around 100–250 F g${}^{-1}$ [3]. Both the electrochemical stability of the electrolyte and the presence of impurities in the carbon electrode limit the operating voltage of EDLC devices [21,22,40]. For example, when using aqueous electrolytes, the operating voltage is limited to just 1.23 V due to the electrolysis of water, in comparison to 3 V or more for common non-aqueous electrolytes [40]. Consequently, another important area of research currently focuses on improving electrolyte stabilities and the purity of carbon electrode materials. Major improvements in the electrochemical performance of supercapacitors have already been achieved through optimisation of the porous structure to improve pore accessibility and increase specific capacitance. This will be discussed in detail in Section 2.5.

**Figure 7:**
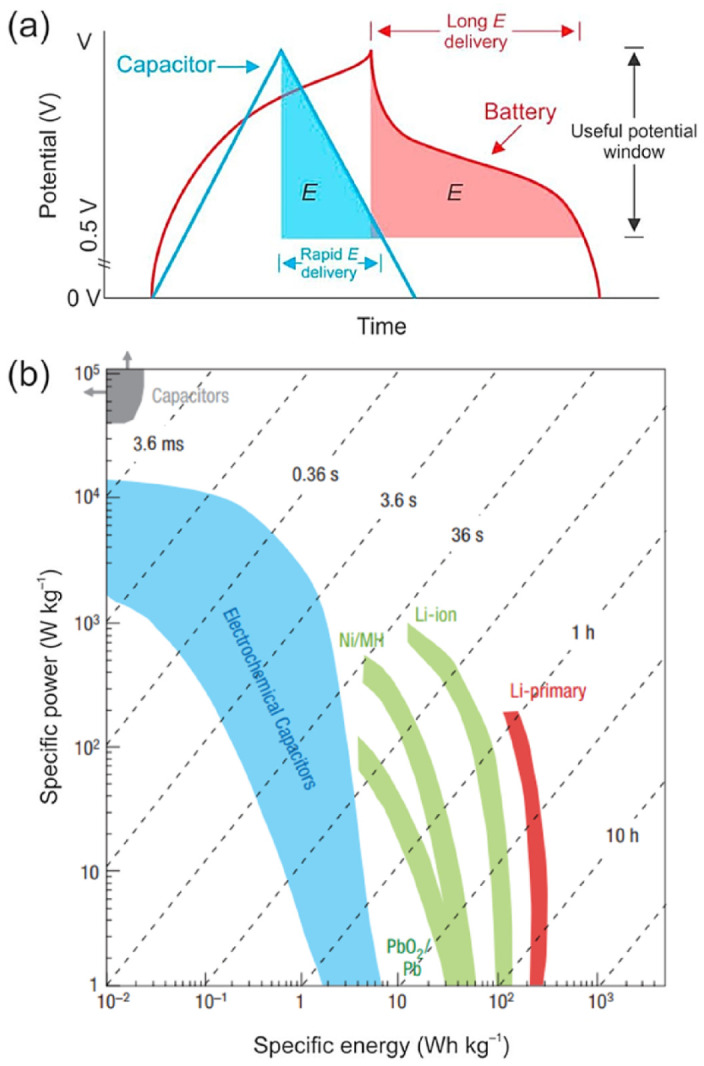
(**a**) Galvanostatic charge-discharge profiles for materials with capacitive and bulk redox (battery-like and pseudocapacitive materials) behaviour and (**b**) Ragone plot showing the energy density vs. the power density for common electrical energy storage devices as well as lines of constant time obtained by dividing the energy density by the power. Reprinted with permission from Ref. [39]. 2019, Elsevier.

Figure 7b shows a Ragone plot, which is the standard method of graphical comparison for energy storage technologies in terms of their energy and power storage capabilities [39]. The origin of the hooked shape seen in the Ragone plot is due to increasing polarisation as higher power demands are made on the device, which diminishes the cell voltage, causing further decreased energy density at higher power densities [3].

The graph confirms the previously mentioned fact that supercapacitors can reach much higher power densities than battery technologies, but struggle to reach comparable energy densities [3]. Commercial EDLCs are only able to reach energy densities in the range of 3–10 Wh Kg${}^{-1}$ [2], in comparison, lithium-ion batteries can reach energy densities of 180 Wh Kg${}^{-1}$ [38]. The difference in energy stored by supercapacitors and batteries can also be seen in the shaded areas of the galvanostatic charge-discharge (GCD) profiles in Figure 7a, with a significantly smaller area present for supercapacitors. This decreases even further when using aqueous electrolytes due to the aforementioned requirements for low operating voltage [39]. Since rapid energy delivery is one of the major advantages of supercapacitor technology, the main way to increase the energy density without majorly affecting this is to find methods to increase the operating voltage window. Current research to improve the energy density of supercapacitors has been focused on functionalisation of the carbon materials, with additions ranging from pseudocapacitive metal oxides to complex carbon structures. Recent progress in functionalised GNP supercapacitors will be discussed in detail in Section 3.2.

A further problem is introduced when considering the industrial-scale production of supercapacitor technologies. Currently, most of the materials used are high cost with energy intensive production methods, particularly during the carbonisation and activation of the carbon electrode materials [2]. Consequently, there is a recent research focus on developing cheap large-scale fabrication processes for carbon nanomaterials, in addition to more environmentally friendly and energy-efficient production methods [54,57].

In addition to improving energy and power densities, future development of supercapacitors should also focus on minimising device size and weight [10]. This is particularly important if supercapacitors are to be included in portable electronic devices, on which much of society now depends. Due to inactive components such as separators, current collectors and packaging contributing mass, in addition to inactive pore space, typically only about 30% of the total supercapacitor mass is active mass [10]. This causes problems, as the active mass ratio should be as high as possible in order to have the best possible energy density. Thus, minimising the device size is beneficial to both practical use and intrinsic properties. Further problems arise when using supercapacitors in harsh environments such as the oil industry, automotive production, and space exploration, where the devices are subject to extreme temperatures or radiation [2,39]. One of the limiting factors for this is the stability of the liquid electrolyte [39], thus more research is needed to solve this, perhaps by improving the utilisation of solid electrolytes.

### 2.5. The Effect of Porous Structure

According to electric double layer (EDL) theory, one of the most crucial factors in achieving a high specific capacitance is for the electrodes to have a large accessible surface area [10]. However, this structural optimisation is also affected by the pore size distribution and accessibility to electrolyte [3]. Thus, the design of the entire porous structure of supercapacitor electrode materials is crucial to their performance. Ideally, to have the best possible pore utilisation and highest specific capacitance, the porous structure should have a hierarchical structure containing macropores (>50 nm) for electrolyte infiltration, mesopores (2–50 nm) for ion transport, and micropores (<2 nm) for charge storage [2,40]. The success of ion migration within the porous matrix depends on both the molecule size and the pore size; if the solvated molecules and ions are much smaller than the pore, then they cannot break the energy barrier to access it [2,60]. Therefore, the size of the ions in the electrolyte must also be taken into consideration when choosing the desired pore size of the electrode material.

The presence of micropores has previously been attributed as a source of capacitance limitation, particularly for ultramicropores (<0.7 nm) since they are smaller than the solvated ion size and were believed to be too small to house the electrolyte ions [38,40]. As they were considered obsolete and believed to provide no contribution to the EDL, many studies into pore engineering were focused on reducing ultramicroporosity in favour of increasing the number of pores in the range of 2 to 5 nm, with the aim of housing two solvated ions with ease [38]. However, this only provided moderate improvements to the capacitances of the electrodes; specific capacitance results had stalled at 100–120 F g${}^{-1}$ for organic electrolytes and 150–200 F g${}^{-1}$ for aqueous electrolytes [38]. In a prominent recent discovery, materials containing micropores were in fact found to have a significant capacitance contribution from the micropores [38,40,61,62,63]. One study found that the efficiency of double-layer formation in microporous activated carbon (AC) was optimal when the average pore-size was around 0.7 nm for aqueous electrolytes and 0.8 nm for organic electrolytes. Gravimetric capacitances were reported in the range of 124 to 286 F g${}^{-1}$ for microporous AC with a range of pore sizes, all averaging on the nano-scale [62]. Partial ion desolvation was suggested as a possible explanation for the high capacitances observed in sub-nanometre pores. This would lead to the ions becoming distorted and therefore being able to reach closer to the carbon wall, hence decreasing the thickness of the EDL, *d*, in Equation (2) [40].

**Figure 8:**
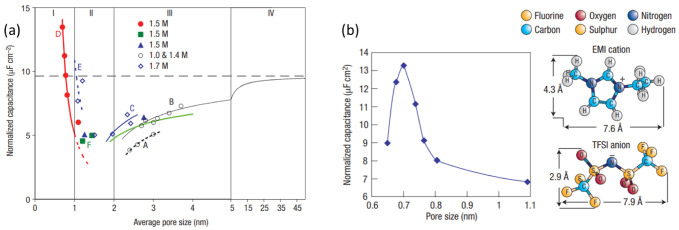
(**a**) Specific capacitance normalised by SSA as a function of pore size for different carbon samples, all tested in the same electrolyte ($NEt_{4}BF_{4}$ in acetonitrile; concentration given in key). The symbols show experimental data (A, B: templated mesoporous carbons; C: activated mesoporous carbons; D, F microporous CDC; E: microporous activated carbon) and the trend lines show model fits [38]. (**b**) Normalised capacitance change as a function of the pore size of CDC samples, with inset showing the structure and size of the electrolyte ions. Adapted with permission from Ref. [38]. 2009, World Scientific.

This theory was confirmed in a study involving carbide-derived carbons (CDCs). These materials allowed for a very narrow and finely tuned pore-size distribution ranging from 0.6 to 1.1 nm to be achieved, with a mean pore size that could be controlled with extreme accuracy [38,61]. The electrochemical properties were analysed for CDC electrodes submerged in a solution of 1 M $NEt_{4}BF_{4}$ in acetonitrile-based electrolyte [61]. The results showed that the normalised capacitance decreased with decreasing pore size until a critical point close to 1 nm was reached, after which the capacitance rose sharply as pore size approached the ion size [61]. Since the electrode materials used were exclusively microporous, the increase in capacitance in the presence of ultramicropores clearly showcased their important contribution. It also confirmed that the distance between the ion and the carbon surface was shorter for ultramicropores, and that the ions must be at least partially stripped of their solvation shell to occupy these pores. This data, along with corroborating data from other studies on a variety of materials, is summarised in Figure 8a [38].

Further studies have supported this theory and concluded that maximum capacitance is obtained when the pore size is in the same range as the ion dimensions, which usually sits on the micropore scale [38]. CDC electrodes in a solvent-free electrolyte of [EMI${}^{+}$, TFSI${}^{+}$] ionic liquid at 60 ${}^{\circ }$C showed the best capacitance with samples with an average pore size of around 0.7 nm. As is shown in Figure 8b, both ions in the electrolyte also had maximum dimensions of around 0.7 nm, suggesting that a single ion per pore gives the highest capacitance [38]. The discovery that ion desolvation occurs in pores which are smaller than the solvated ions has led to large increases in specific capacitance for EDLCs, and has opened the door to designing high energy density devices [38].

## 3. Recent Progress in GNP Supercapacitors

Table 1 summarises recent developments in GNP-based supercapacitors, specifying the operating conditions and structure used, and the results obtained. There has been a wide range of different approaches to improving the properties of supercapacitors using GNPs in recent years. Some studies have focused on altering the structure of plain GNPs in order to optimise it to specific operating conditions to achieve the highest possible results [21,51,55,64]. Others have explored functionalisation using various moieties in order to improve the energy density by incorporating pseudocapacitance into the devices [52,53,65,66]. Further research has explored how GNPs and functionalised GNP materials can be manufactured using novel methods, to lower the cost or environmental impact of the process [52,54,57,58].

### 3.1. Plain GNPs

Plain GNPs have shown relatively high capacitances and energy densities, even without additional structures or functionalisation. Commercially obtainable GNPs have shown a capacitance of 70 F g${}^{-1}$ at a scan rate of 25 mV s${}^{-1}$ in 1 M $TEABF_{4}$/acetonitrile solution, alongside a maximum power and energy density of 5.45 kW kg${}^{-1}$ and 13 Wh kg${}^{-1}$, respectively, [51]. This was a better electrochemical performance than a commercial AC electrode, which demonstrated a specific capacitance of 40 F g${}^{-1}$ and energy density of 4.47 Wh kg${}^{-1}$.

**Figure 9:**
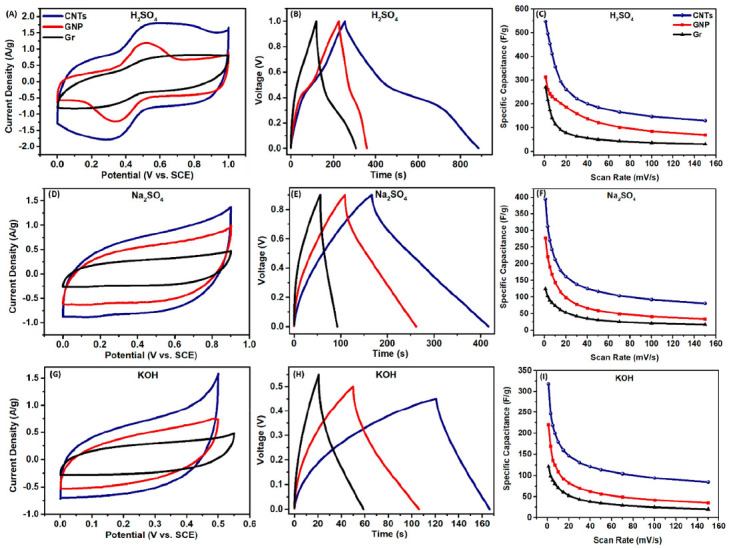
Performance of the carbon materials in the positive potential window in different electrolytes measured in three-electrode configuration: (in $H_{2}SO_{4}$ electrolyte) (**A**) CVS of carbon materials at 5 mV s${}^{-1}$, (**B**) GCDs of carbon materials at 0.7 A g${}^{-1}$, (**C**) rate capability of the carbon materials; (in $Na_{2}SO_{4}$ electrolyte) (**D**) CVS of carbon materials at 5 mV s${}^{-1}$, (**E**) GCDs of carbon materials at 0.7 A g${}^{-1}$, (**F**) rate capability of the carbon materials; (in KOH electrolyte) (**G**) CVS of carbon materials at 5 mV s${}^{-1}$, (**H**) GCDs of carbon materials at 0.7 A g${}^{-1}$, (**I**) rate capability of the carbon materials. Reprinted with permission from Ref. [21]. 2021, ACS.

**Figure 10:**
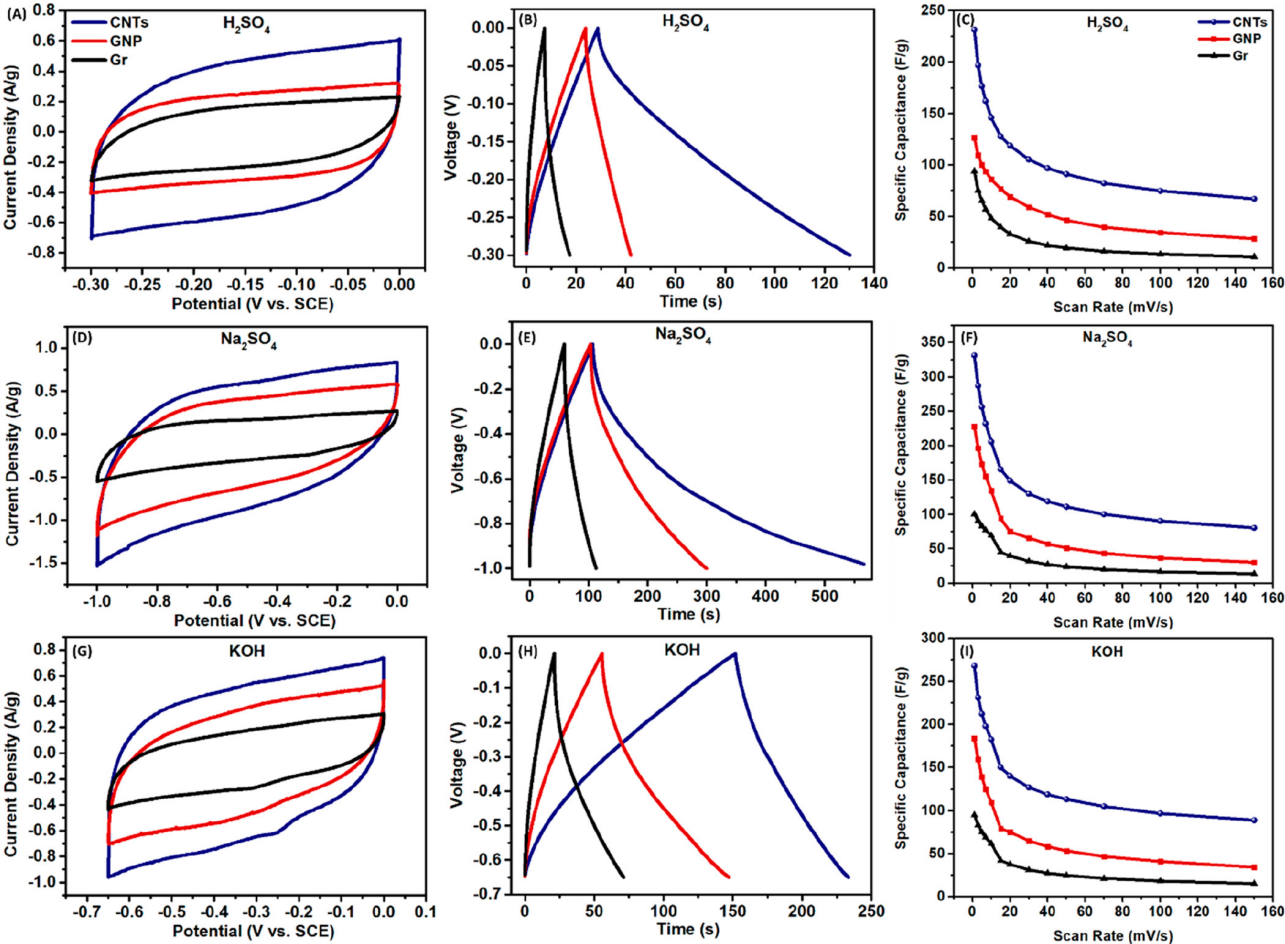
Performance of the carbon materials in the negative potential window in different electrolytes measured in three-electrode configuration: (in $H_{2}SO_{4}$ electrolyte) (**A**) CVS of carbon materials at 5 mV s${}^{-1}$, (**B**) GCDs of carbon materials at 0.7 A g${}^{-1}$, (**C**) rate capability of the carbon materials; (in $Na_{2}SO_{4}$ electrolyte) (**D**) CVS of carbon materials at 5 mV s${}^{-1}$, (**E**) GCDs of carbon materials at 0.7 A g${}^{-1}$, (**F**) rate capability of the carbon materials; (in KOH electrolyte) (**G**) CVS of carbon materials at 5 mV s${}^{-1}$, (**H**) GCDs of carbon materials at 0.7 A g${}^{-1}$, (**I**) rate capability of the carbon materials. Reprinted with permission from Ref. [21]. 2021, ACS.

An in-depth study which compared commercial carbon materials in different electrolytes presented a road map for the proper selection of the carbon material for supercapacitor electrode use [21]. The road map was based on a systematic investigation of the behaviour of CNTs, GNPs and graphite in the most commonly used acidic, neutral and basic aqueous electrolytes (0.5 M $H_{2}SO_{4}$, 0.5 M $Na_{2}SO_{4}$ and 0.5 M KOH) using both computational and electrochemical tools. Various potential windows were used depending on the electrolyte, with both negative and positive potential windows explored. The results for both the positive and negative and negative potential windows can be seen in Figure 9 and Figure 10, respectively. The study concluded that the proper choice of carbon material with the complementary electrolyte and potential window can be used to design more efficient electrode materials for energy storage [21].

Pseudocapacitive and battery like materials are heavily electrolyte dependent, as they are only electrochemically active in specific electrolytes. Therefore, the roadmap stated that the electrolyte characteristics should be taken into account when choosing the faradaic or pseudocapacitive functionalisation material. The charge storage mechanism was found to be electrolyte dependent, and potential window capability varied depending on the electrolyte. $Na_{2}SO_{4}$ exhibited the best potential window performance with −1 to 0.9 V, whereas the positive electrodes in $H_{2}SO_{4}$ delivered the best capacitance. $H_{2}SO_{4}$ enabled keto-enol tautomerism in the positive potential window and succeeded in enlarging the potential window to 1 V. It is important to use the correct potential window, as an overestimated potential window can affect the device stability and an underestimated window can affect the achieved power density. Quantum capacitance calculations were used to help identify the reasoning behind the differing performances between the positive and negative potential windows. Density functional theory (DFT) was used to understand the reasons behind the EDL capacitance through calculation of the quantum capacitance of the carbon electrode materials. DFT was chosen as it has proven to be a good tool for predicting the properties of energy materials. Quantum capacitance demonstrates the electronic response of the electrode material under an applied voltage. Thus, a higher quantum capacitance indicates higher levels of EDL behaviour in the electrode material. This was used to investigate the EDL performance in various potential windows to develop a theoretical comparison for the practical data [21]. The surface area of the GNPs used was found to be 125.9 m${}^{2}$g${}^{-1}$, using BET fitting for nitrogen adsorption/desorption. The GNP material was microporous with a large pore size peak at around 2-3 nm, and microporous surface area accounting for 19.4% of the total area. The electrodes used a graphene sheet as the current collector to avoid any additional EDLC contributions and provide more accurate results of the real performance of the carbon materials. The carbon ink was prepared using 90% active material, 10% polyvinylidene fluoride (PVDF), and dimethylformamide solvent.

In the positive potential window, the carbon materials all exhibited clear redox behaviour in the $H_{2}SO_{4}$ electrolyte and EDL behaviour in the $Na_{2}SO_{4}$ and KOH electrolytes, as is evident in Figure 9. In comparison, the carbon materials presented EDL behaviour in all three electrolytes in the negative potential window, as shown in Figure 10 [21]. In all combinations, the GNP electrodes showed higher specific capacitance than the graphene electrodes and lower capacitance than the CNT electrodes. $H_{2}SO_{4}$ provided the largest positive potential window of 1 V, whereas $Na_{2}SO_{4}$ provided the largest negative potential window of −1 V. In the electrolyte comparison, the specific capacitance followed the pattern of $H_{2}SO_{4}$ > $Na_{2}SO_{4}$ > KOH in the positive potential for all samples and $Na_{2}SO_{4}$ > KOH > $H_{2}SO_{4}$ in the negative potential window. This may be attributed to the width of the potential window provided by each electrolyte, alongside the binding affinity and diffusion of the ionic species at different potentials. The GNP electrodes scanned at 5 mV s${}^{-1}$ in $H_{2}SO_{4}$, $Na_{2}SO_{4}$ and KOH displayed specific capacitances of 242.98 F g${}^{-1}$, 190.70 F g${}^{-1}$ and 135.78 F g${}^{-1}$, respectively, in the positive potential windows. Meanwhile, specific capacitances of 100.34 F g${}^{-1}$, 173.46 F g${}^{-1}$ and 139.28 F g${}^{-1}$, respectively, in the same electrolytes for the negative potential windows [21].

**Figure 11:**
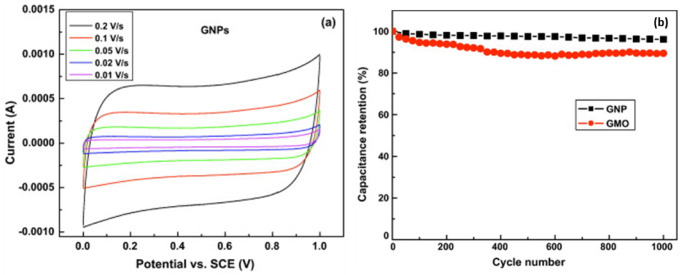
(**a**) Cyclic voltammetry results of GNPs at various scan rates in 2 M KCl aqueous electrolyte, and (**b**) Variation of the specific capacitance of GNP and GMO ($MnO_{2}$-GNP) from cyclic voltammetry at scan rate of 400 mV/s in 2 M KCl aqueous electrolyte. Adapted with permission from Ref. [52]. 2011, Elsevier.

Non-commercial GNPs, synthesised from reduced graphene oxide (rGO), have also shown similarly impressive electrochemical properties [52]. Specific capacitance of 100–150 F g${}^{-1}$ at 0.01–0.2 V s${}^{-1}$ was obtained from CV analysis and 125 F g${}^{-1}$ at 1 A g${}^{-1}$ from the charge-discharge curve, for rGO-synthesised GNP in 2 M KCl electrolyte, in addition to energy density measurements of 13.9–20.8 Wh kg${}^{-1}$. The GNPs also showed a very impressive 92% capacitance retention after 100 cycles at 400 mV/s and had a quasi-rectangular CV curve, as visible in Figure 11, indicating ideal EDLC behaviour [52]. As with single-layer graphene, there is a large barrier to reach a simple, efficient, cost-effective and scalable production method [54].

This problem was recently investigated in a study that used graphene nanoplatelets manufactured using a novel large-scale manufacturing technique which utilised airless high-pressure spray exfoliation to manufacture the graphene nanoplatelets [57]. Symmetric supercapacitors were made using the GNPs, which had 5 to 8 layers, and were tested in both organic (1 M $TEABF_{4}$/acetonitrile solution) and aqueous electrolytes (1 M $Na_{2}SO_{4}$). Maximum specific capacitances of 26 F g${}^{-1}$ at 6 A g${}^{-1}$ (their lowest measured current density) and 86 F g${}^{-1}$ at 2 A g${}^{-1}$ were obtained in the organic and aqueous electrolytes, respectively. For the organic electrolyte, 33% of the capacitance was retained at 14 A g${}^{-1}$ compared to 6 A g${}^{-1}$. The highest organic energy density measured was 12.2 Wh kg${}^{-1}$, when the power density was 5 kW kg${}^{-1}$ at a current density of 6 A g${}^{-1}$. The highest organic power density reached was 12.65 kW kg${}^{-1}$ at 14 A g${}^{-1}$, however the energy density had dropped to 3.8 Wh kg${}^{-1}$ at this current density. Capacitance retention after cycling 5000 cycles in the organic electrolyte at 14 A g${}^{-1}$ was 90% while coulombic efficiency remained high, at 84% after cycling. When tested in the aqueous electrolyte, the electrode had a capacitance retention of 38% at 15 A g${}^{-1}$ compared to 2 A g${}^{-1}$. The highest aqueous energy density measured was 11 Wh kg${}^{-1}$, when the power density was 1 kW kg${}^{-1}$ at a current density of 2 A g${}^{-1}$. The highest aqueous power density reached was 75. kW kg${}^{-1}$ at 15 A g${}^{-1}$, however, the energy density had dropped to 3.5 Wh kg${}^{-1}$ at this current density. Capacitance retention after cycling 5000 cycles in the $Na_{2}SO_{4}$ at 12 A g${}^{-1}$ was 93% while coulombic efficiency remained high, at 93% after cycling [57].

**Figure 12:**
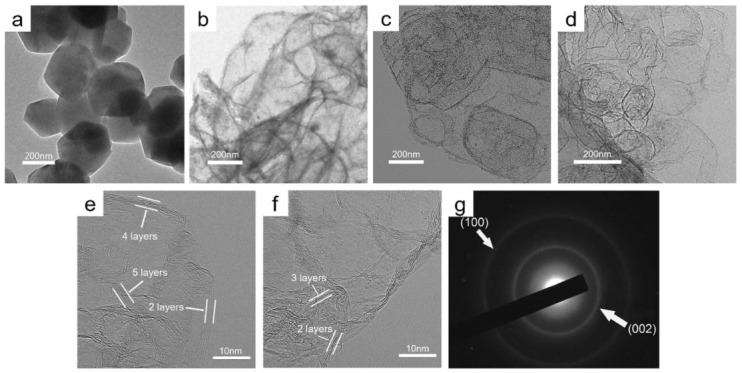
TEM images of (**a**) MgO template, (**b**) SHS-4, (**c**) SHS-8, (**d**) SHS-10, HR-TEM graph of (**e**) SHS-8, (**f**) SHS-10, and (**g**) selected area diffraction (SAED) pattern of SHS-8. Samples labelled SHS-n according to the mass ratio of Mg/MgO in the self-propagating high temperature combustion synthesis samples, which is 1:4, 1:8 and 1:10, respectively. Reprinted with permission from Ref. [58]. 2022, Elsevier.

Other innovative formation methods of GNP particles have also been explored, such as a novel process of a self-propagating high-temperature combustion synthesis reaction between CO and magnesium metal, using MgO powder as the deposition template [58]. This resulted in a few-layered graphene material forming, which mimicked the morphological features of the MgO powder, as can be seen in Figure 12. The structures had 2–6 graphene layers and surface areas ranging from 451 to 928 m${}^{2}$g${}^{-1}$ depending on the manufacturing conditions. The GNPs were formulated into an ink using PVDF, NMP and carbon black before the ink was printed onto aluminium foil to create electrodes. A symmetrical supercapacitor was assembled using the electrodes and EMI [TFSI] ionic liquid as the electrolyte. The highest specific capacitance achieved using the material was 222 F g${}^{-1}$ at 1 A g${}^{-1}$, using the sample with the highest surface area. The sample with mid-range surface area was able to achieve both a relatively high energy density and power density simultaneously, reaching 76.3 Wh kg${}^{-1}$ at 1.75 kW kg${}^{-1}$. It also succeeded in retaining the energy density at a relatively high level of 48.6 Wh kg${}^{-1}$ at the maximum recorded power density of 35 kW kg${}^{-1}$. A positive relation was found between the specific capacitance and surface area, as would be expected. The capacitance retention was measured to be 70% from 1 A g${}^{-1}$ to 10 A g${}^{-1}$, and the cycling stability was very good, with 99% of the specific capacitance retained after 8000 cycles at 10 A g${}^{-1}$ [58].

Flexible devices are a newer field of study, which requires different materials and techniques to traditional supercapacitors. Recently developed flexible GNP supercapacitors using a gel electrolyte of polyvinyl alcohol (PVA) and phosphoric acid ($H_{3}PO_{4}$) were successful in reaching comparable results to non-flexible devices [67]. The design of the supercapacitors was symmetrical with a paper/$BaTiO_{3}$ gel coated separator. CV measurements gave values of 380 F g${}^{-1}$ and 79 F g${}^{-1}$ for the devices at 20 mV s${}^{-1}$ and 150 mV s${}^{-1}$, respectively. In comparison, a slightly lower specific capacitance of 163 F g${}^{-1}$ was obtained via GCD analysis at 0.06 mA cm${}^{-2}$. The highest measured energy density for the GNP supercapacitor was 79 Wh Kg${}^{-1}$. The device also showed good cycling stability after 2000 charge/discharge cycles, however, the study gave no details on the cycling parameters or results [67].

Aside from the functionalisation that will be discussed in Section 3.2, all-carbon composites can also be used to improve the electrochemical properties of GNPs [68,69,70]. A novel Fe-based catalysis method was used to build hollow carbon spheres in which the shell comprised of a 2D microporous carbon and FLG heterostructure [64]. The aim of the nano-sized thickness of the shell was to increase the exposure of the micropores, improve the conductivity and shorten the ion transport distance. The inclusion of meso- and macropores further aimed to improve the ion adsorption of the high density open micropores. The material showed a maximum specific capacitance 128 F g${}^{-1}$ at 5 mV s${}^{-1}$ when measured in a three-electrode system in 2 M KOH electrolyte. The capacitance retention was lower than most non-composites, at 71% for the high scan rate of 200 mV s${}^{-1}$. The high specific capacitance was attributed to the short micropore channels and high-density of exposed ion adsorption sites in the ultrathin microporous carbon layer in the heterostructure. The specific capacitance of the material was further improved through the use of selective KOH etching to create meso- and micropores in the carbon. This greatly increased the measured surface area for the samples, from 615 m${}^{2}$g${}^{-1}$ pre-etching to 970 m${}^{2}$g${}^{-1}$ post etching, with 91% microporosity. The increase in microporosity and surface area confirmed that the etching process could be used to improve the connection of open micropore channels in the structure. The etching treatment succeeded in improving the specific capacitance; the maximum measured specific capacitance increased to 187 F g${}^{-1}$ at 5 mV s${}^{-1}$, with a capacitance retention of 84% at 200 mV s${}^{-1}$.

### 3.2. Functionalised GNP Supercapacitors

Functionalisation and surface treatment of carbon materials can be used to improve and alter a variety of electrode properties. Depending on the functional group and functionalisation method, different properties can be affected including improvements to the specific capacitance, energy and power density, hydrophobicity and wettability, or the addition of pseudocapacitive components [53,71,72,73]. However, functionalisation can also produce undesirable side effects such as gassing during operation, faster self-discharge and reduced stability, particularly at higher temperatures [11], so the surface groups must be chosen carefully. Due to the smaller size of the research field for GNP-based supercapacitors currently relative to other carbon nanomaterials such as graphene and CNTs, there are far fewer studies into functionalised GNP electrodes. However, in addition to the available research on functionalised GNPs, studies on other high-performing functionalised carbon nanomaterials are useful in informing future GNP research as the effect on the properties are likely to translate similarly from these materials to GNPs.

#### 3.2.1. Pseudocapacitive Functionalisation

One common method of introducing pseudocapacitance to carbon materials is through heteroatom doping, which enhances the charge capability by opening the intrinsic band gap and offering more active sites [2,74,75,76,77,78,79,80,81,82,83,84]. Methods to create these heterodoped materials include chemical vapour deposition, pyrolysis with a hetero precursor and self-doping [2]. Pseudocapacitance can also be added through polymer functionalisation, with polyaniline (PANI) being one of the most commonly used polymers [85,86,87,88,89,90,91,92].

**Figure 13:**
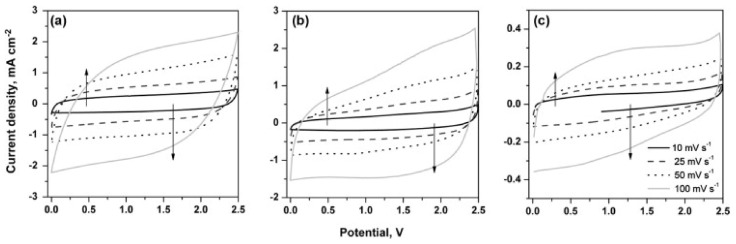
CV curves at various scan rates for the electrodes based on: (**a**) commercial GNP, (**b**) $MnO_{2}$-GNP and (**c**) GNP exfoliated from graphite. The slight redox peak visible in (**b**) shows the very limited pseudocapacitance that was achieved with the $MnO_{2}$-functionalised GNPs. Reprinted with permission from Ref. [51]. 2015, Elsevier.

Alternatively, specific capacitance may be increased by hybridising the EDLC with metal oxides or hydroxides such as $RuO_{2}$, $MnO_{2}$ or $Fe_{2}O_{3}$ with the main aim of increasing the specific capacitance by increasing the proportion of pseudocapacitance present [2,93,94]. $MnO_{2}$ is currently considered the most promising metal oxide due to its low cost and ease of manufacturing [2]. Unfortunately, due to limited charge transfer kinetics, poor electronic conductivity and poor electrolyte ion penetration metal oxide composites generally exhibit much lower specific capacitance than theoretically expected. The maximum practically achievable capacitance of $MnO_{2}$ is far lower than the theoretical maximum of 1100 F g${}^{-1}$[52]. One study reported the functionalisation of GNPs with $MnO_{2}$ in order to increase the material’s energy density through the increase in the pseudocapacitive properties [51], using commercial GNPs dispersed in a 0.01 M $KMnO_{4}$ solution, followed by dropwise addition of 0.05 M citric acid until the solution became brown-purple in colour. The solution was then heated to 80 ${}^{\circ }$C in a distillation column with full reflux for 7 h, before the functionalised GNPs were filtered out and dried at 80 ${}^{\circ }$C for 12 h. However, the surface area only reached 39 m${}^{2}$g${}^{-1}$ in comparison to 479 m${}^{2}$g${}^{-1}$ for GNP and 1000 m${}^{2}$g${}^{-1}$ for AC. Furthermore, the intended pseudocapacitive effects were not seen, with specific capacitance only reaching 27.5 F g${}^{-1}$ which was less than half that of the plain GNPs, and only a slight pseudocapacitive element visible in the CV curve in Figure 13b. This was attributed to the lower amount of GNPs present in the electrode due to functionalisation, so that the intended pseudocapacitance was not activated with the chosen electrolyte [51].

**Figure 14:**
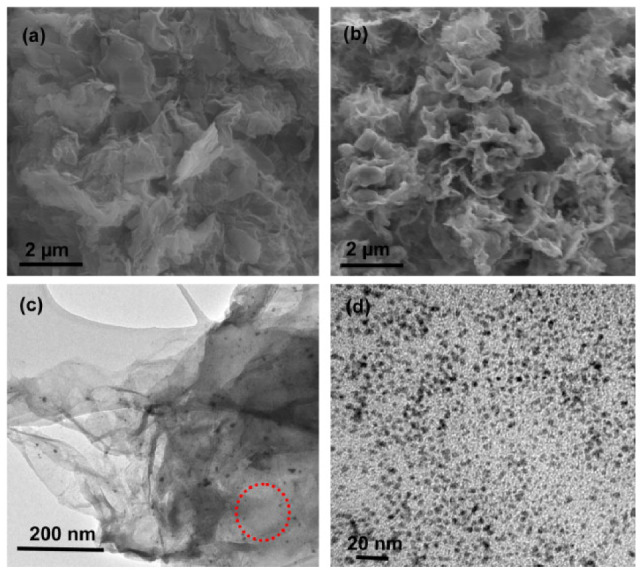
(**a**) SEM image of GNPs before incorporated with MnO2; (**b**) SEM image of $MnO_{2}$-GNP composite; (**c**) TEM image of $MnO_{2}$-GNP composite at low magnification; (**d**) TEM image of the area highlighted by the dashed cycle in (**c**) at high magnification. Reprinted with permission from Ref. [52]. 2011, Elsevier.

Another study succeeded in creating a structure containing a homogeneously distributed $MnO_{2}$ nanoparticle coating on the surface of GNPs [52]. This study utilised GNPs synthesised from rGO instead of commercial GNPs, but did use the same method to functionalise the GNPs as was just detailed above. Figure 14 shows SEM and TEM imaging of the GNPs and $MnO_{2}$-GNPs, with the homogeneous coating of $MnO_{2}$ nanoparticles with minimal aggregation clearly presented in Figure 14d. When electrochemical measurements were made using 2 M KCl as the electrolyte, a specific capacitance of 450–542 F g${}^{-1}$ at 0.01–0.2 V s${}^{-1}$ was measured from the CV curve and 511 F g ${}^{-1}$ at 1 A g${}^{-1}$ from the charge-discharge curve. Therefore, the functionalisation had succeeded in increasing the capacitance to around 4 times that of the plain GNPs. An increase in energy density was also noted, increasing from 13.9 to 20.8 Wh kg${}^{-1}$ for the unmodified GNPs to 62.5–75.3 Wh kg${}^{-1}$. The cycle life did decrease slightly, from 92% to 85% after 100 cycles at 400 mV s${}^{-1}$, though this was likely only a result of the increased role of pseudocapacitance as the CV curve did show that there was a combination of pseudocapacitance and EDLC behaviour present [52]. However, the material did have good rate capability, with 83% of the capacitance retained even at a high scan rate of 1000 mV s${}^{-1}$.

#### 3.2.2. Carboxyl Functionalisation

Pure graphene structures often suffer from hydrophobicity which limits the accessibility of electrolyte ions to the micropores and decreases the electroactive surface area, thus decreasing the attainable specific capacitance [53]. It has been well documented that giving CNTs an oxidation treatment with a strong acid like $H_{2}SO_{4}$ or $HNO_{3}$ introduces oxygen-containing functional groups such as carboxyl groups onto the structure [7,95,96,97]. A previous study showed that the hydrophilicity of multiwalled CNTs in aqueous $H_{2}SO_{4}$ electrolyte was improved by the introduction of surface carboxyl groups, leading to a 3.2 times larger capacitance of 51.3 F g${}^{-1}$ [95]. Besides the increase due to the increased wettability, the carboxylated multiwalled CNTs also demonstrated an increase in capacitance attributed to raised pseudocapacitance, governed by redox reactions of the oxygen functionalities on the carbon surface [39,95].

As an alternative to bulk-functionalisation, graphene-based materials can undergo edge functionalisation, in which chemical moieties are selectively attached to the edges of graphene sheets while leaving the bulk of the plane undamaged [1]. This imparts some characteristics of the attached moieties into the carbon, while allowing the graphene to largely preserve its original physicochemical properties. This can therefore improve the solubility, film-forming properties, electroactive surface area, hydrophilicity or electrocatalytic activity [1,53]. As there is minimal serious deterioration of the intrinsic graphene properties, more effective ion adsorption and rapid electrolyte diffusion within the pores is possible than in bulk functionalised materials [53]. Edge functionalisation is notably interesting due to the utilisation of dangling bonds at the edge of graphene sheets, which have been shown to be more reactive than the covalently bonded carbon in the bulk plane [1].

**Figure 15:**
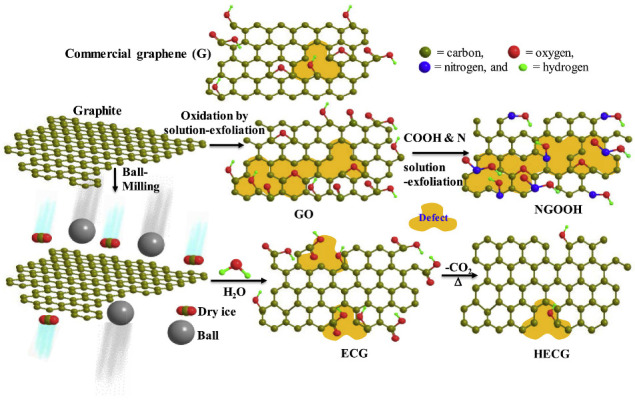
Schematics of the synthesis of nitrogen-doped carboxylic graphene (NGOOH), edge-carboxylated graphene nanoplatelets (ECG), and heat-treated edge-carboxylated graphene nanoplatelets (HECG). Reprinted with permission from Ref. [53]. 2019, Elsevier.

A recently developed approach, which could be used for the simple mass production of edge-carboxylated graphene nanoplatelets, involves ball milling graphene with dry ice to produce surface-carboxylated graphite particles [1,27,28,30,98,99]. This ball-milling method is more environmentally friendly than conventional edge-functionalisation methods that involve the solution-exfoliation of graphite [30,53,99]. Figure 15 illustrates the synthesis mechanism for the edge-carboxylated GNPs. During the milling, the functional groups are incorporated onto the surface of broken graphite particles, due to the mechanochemical cracking of the graphitic C-C bonds. When dispersed in a polar solvent such as water, large repulsive forces arise between the surface carboxylates and the polar solvent, causing dissolution exfoliation of the graphite particles and forming edge-carboxylated FLG [27,28,98].

This method has been successfully employed to create edge-carboxylated graphene nanoplatelets with only around 16.2% structural defects, in comparison to around 48.9% found in nitrogen-doped carboxylic graphene synthesised via the traditional method of solution-exfoliation shown in Figure 15 [53]. The material also contained a higher concentration of edge-carboxylic groups in comparison to carboxylic graphene made using the conventional method. To create the edge-carboxylated GNPs, 5 g pristine graphite and 100 g of dry ice were ball-milled at 500 rpm for 48 h before being treated with a 1 M aqueous HCl solution in order to remove metallic impurities [53]. The resultant edge-carboxylated GNPs appeared to form less aggregation and smaller pores, indicating that the carboxylic groups repelled each other and reduced the restacking. Moreover, an extremely high capacitance of 365.7 F g${}^{-1}$ was observed when the edge-carboxylated GNPs were analysed using a 1 M $H_{2}SO_{4}$ electrolyte; measured for a sample with 8.4% edge-carboxylic groups and 16.2% structural defects. To allow for a more practical comparison to real device performance, 2-electrode symmetric cell performance was also studied. This gave a specific capacitance of 284.1 F g${}^{-1}$ at 1 A g${}^{-1}$ and energy density of 25.25 Wh kg${}^{-1}$ [53].

#### 3.2.3. Hydroxyl Functionalisation

**Figure 16:**
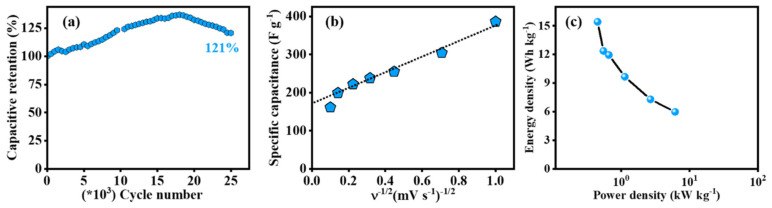
Supercapacitor performance of HRFG-96 in 2 M $H_{2}SO_{4}$: (**a**) long-term cyclic stability test for 25,000 cycles at 100 mV s${}^{-1}$, (**b**) specific capacitance vs. inverse of the square root of the scan rate, and (**c**) Ragone plot in two-electrode configuration. Reprinted with permission from Ref. [66]. 2022, ACS.

Hydroxyl groups are another moiety which have recently been explored to improve supercapacitor electrode material properties [100]. In a recent study [66], covalently functionalised hydroxyl-rich FLG was synthesised via a time-dependent 1,3 dipolar cycloaddition reaction of azomethine ylide containing hydroxyl groups with defect-rich oxygen-functionalised few-layer graphene. Characterisation found the material to be mesoporous, with a narrow pore size range of 3–5 nm, and a surface area of 187 m${}^{2}$g${}^{-1}$. The material performed very well as a supercapacitor material, providing a specific capacitance of 389 F g${}^{-1}$ at 1 mV s${}^{-1}$ and 320 F g${}^{-1}$ at 1 A g${}^{-1}$ in 2 M $H_{2}SO_{4}$. The material showed a capacitance retention of around 58% at the highest current density of 10 A g${}^{-1}$ and maximum energy and power densities of 15.4 Wh kg${}^{-1}$ and 6.1 kW kg${}^{-1}$, respectively. Analysis found nearly 53% of the specific capacitance to be due to pseudocapacitance resulting from excess hydroxyl functionalisation. Moreover, as a result of enhanced wettability during cycling lowering the charge transfer resistance and improving charge transport, the material exhibited excellent cycle stability of 121% after 25,000 cycles at 100 mV s${}^{-1}$. This impressive performance is shown in Figure 16a. CV analysis at various points throughout the cycling suggested that the increase in specific capacitance was due to two factors: a faster electron transfer rate at the electrode-electrolyte interface during late-stage cycling, and development of enhanced wettability to a significant improvement in active site accessibility and lower charge transfer resistance [66].

#### 3.2.4. Other Functionalisations

Other methods of reducing the hydrophobicity and agglomeration of GNPs when dispersed in liquid have been investigated aside from the aforementioned carboxyl and hydroxyl functionalisation. For example, water-soluble graphene nanoplatelets (WSGNP) were synthesised as a potential method of creating a highly concentrated aqueous colloidal solution [50]. Pretreatment of the GNP powder was carried out through dispersal in a solution containing 0.67 M sodium hypochlorite (NaClO) and 0.67 M sodium bromide (NaBr). After dispersion, the solution was centrifuged prior to dialysis in pure water for 10 days to remove the residual ionic chemicals and extract the finished WSGNPs. Analysis of the WSGNP dispersed in pure water showed smaller agglomerations (3.66 ± 1.73 μm) than plain untreated GNPs (12.95 ± 7.57 μm), suggesting that the pretreatment was successful [50]. The material was briefly tested on electrodes as a flexible freestanding WSGNP film, formed via vacuum filtration of the colloidal solution. A full electrochemical analysis was not carried out, however, the film did demonstrate an excellent conductivity of 2.2 × 10${}^{3}$ to 3.0 × 10${}^{3}$ S m${}^{-1}$; double that of previously reported values for water-soluble graphene [50]. Thus, the WSGNP could prove a promising electrode material and could aid easier formulation of carbon inks. Nevertheless, further work is required to provide a full electrochemical assessment and determine its suitability for supercapacitors.

**Figure 17:**
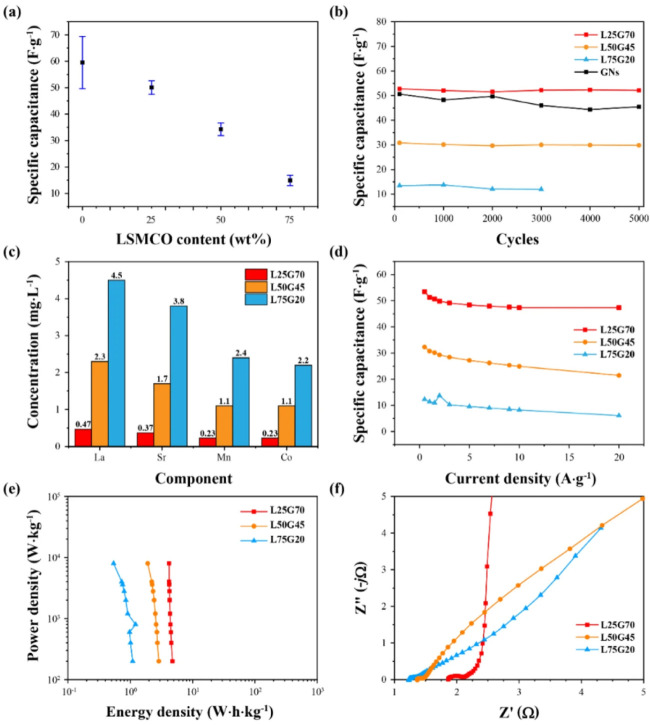
(**a**) Initial specific capacitance according to the LSMCO content (wt%) at the scan rate of 5 mV s${}^{-1}$. Markers and error bars represent the average and standard deviation, respectively, (**b**) The specific capacitance of L25G70, L50G45, L75G20, and GNPs after the cycling test at 20 mV s${}^{-1}$, confirming the electrochemical stability of the LSMCO-based electrodes, (**c**) Metal concentrations dissolved in 1 M $H_{2}SO_{4}$ electrolyte from LSMCO-based electrodes, as measured using inductively coupled plasma-optical emission spectrometry (ICP-OES), (**d**) Variation of the specific capacitance with the current density for all electrodes, (**e**) Ragone plots, and (**f**) Nyquist plots of all electrodes. Reprinted with permission from Ref. [65]. 2022, Nature.

A recent investigation fabricated functionalised supercapacitor electrodes by integrating a perovskite material (La${}_{0.8}$Sr${}_{0.2}$Mn${}_{0.5}$Co${}_{0.5}$O${}_{3-\delta }$, LSMCO) with graphene nanoplatelets, with the aim of integrating the redox reaction capability of the perovskite with the electronic properties of the GNPs [65]. An ABO${}_{3}$-type perovskite was chosen due to their structural stability, providing good potential as pseudocapacitor materials for electrochemical applications [65,101]. The GNPs induced structural distortion within the LSMCO matrix, creating abundant anion vacancies that served as robust charge storage sites. Although the specific capacitance of the perovskite materials was slightly lower, they exhibited much more stable cycling behaviour than previously studied conductive polymers and metal oxides, as is visible in Figure 17b. Electrodes were fabricated using the composite, with carbon paper as the substrate and an ink binder composed of polyvinylidene fluoride (PVDF) and dimethyl sulfoxide (DMSO). Electrochemical characterisation in the stable voltage window of 0 to 0.8 V, using a three-electrode cell with 1 M $H_{2}SO_{4}$, found the average specific capacitance of the composite electrode to be 50.11 F g${}^{-1}$ at 5 mV s${}^{-1}$. The energy density was in the range 4.74–4.20 Wh kg${}^{-1}$ while the current density varied between 0.5 and 20 A g${}^{-1}$ [65]. The full graphical results are displayed in Figure 17, in which it is clear that the electrodes were not only stable during cycling, but also stable when analysed at a range of current densities up to 20 A g${}^{-1}$ [65].

In addition to compositing carbon materials with pseudocapacitors, it is possible to create all-carbon composites by embedding carbon nanomaterials into a carbonaceous backbone [8,64,70,102,103,104,105]. For example, embedding carbon quantum dots into the pores of activated carbon can lead to a specific capacitance three times higher than that of unmodified activated carbon [106]. An all-graphene asymmetric supercapacitor recently reported in a study was constructed from a chemically functionalised graphene cathode paired with either thermally reduced graphene oxide (FG//TrGO) or iodine-doped graphene (FG//IG) as the anode [107]. The cathode was functionalised via the introduction of carbonyl groups on the surface of rGO, and the two asymmetric devices were made to compare the efficacy of thermally reduced graphene versus iodine-doped graphene as the anode. The FG//IG system delivered a capacitance of 228.2 F g${}^{-1}$ at 0.5 A g ${}^{-1}$ in an optimised working potential of 0 to 1.7 V, with a cycle stability of 76% after 4000 cycles. Furthermore, the device showed an energy density of 91 Wh kg${}^{-1}$ at a power density of 424.95 W kg${}^{-1}$. The FG//TrGO system did not perform as highly, but still provided a capacitance of 170.8 F g${}^{-1}$ at 1 A g${}^{-1}$ in an optimised working potential of 0 to 1.3 V, with a cycle stability of 65% after 4000 cycles [107]. The energy density delivered was also lower, with 40.08 Wh kg${}^{-1}$ at a power density of 649.96 W kg${}^{-1}$. Based on these results, it is clear that the FG//IG system showed better electrochemical performance and better potential as a supercapacitor than the FG//TrGO system. This is likely due to pseudocapacitance introduced by the iodine dopant in the anode.

## 4. Conclusions and Perspectives

**Figure 18:**
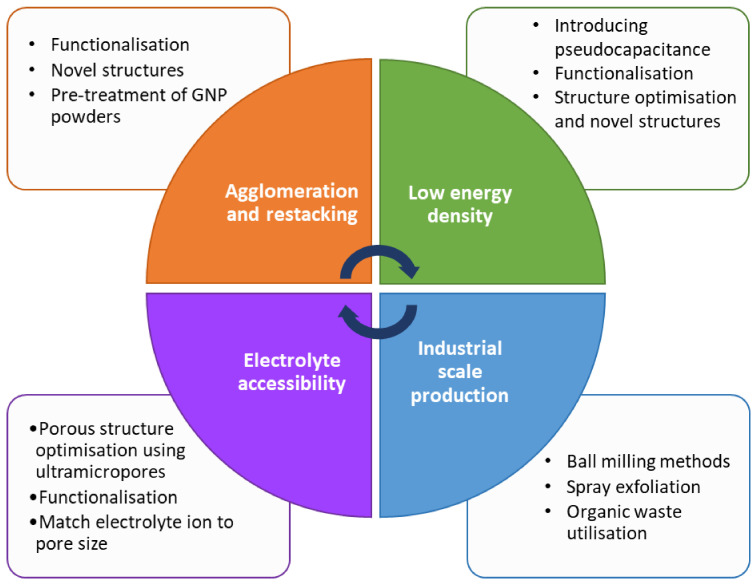
Summary diagram showing the main challenges facing GNP supercapacitors in the centre, and the possible solutions for each respective challenge around the outside.

Supercapacitor design has come far since Becker’s first prototype, and though the energy densities still do not match those of batteries, the performance gap is closing. This has been particularly led by the discovery of the sub-nanometre pore contribution to the capacitance, which has opened a new design field of porous structures. It is now believed that the highest capacitances can be achieved when the pore sizes are similar to the solvated ion dimensions. Graphene-based materials, including graphene nanoplatelets have shown great potential for successful electrode materials and have seen great steps forward in their performance in recent years. Their advantageous properties include very high surface area, chemical and thermal stability and good conductivity; all of which can be tuned as desired through altering the porous structure. However, they are currently not suitable for industrial-scale production of supercapacitors due to their high cost and energy-intensive production methods. The main challenges still facing GNP supercapacitor design are summarised in Figure 18.

There are many areas in which supercapacitors still require improvement, most of which are tied to the challenges described above. Research into these challenges has identified many potential solutions to overcome them, which are shown in Figure 18, many of which revolve around functionalisation of the carbon nanomaterials. The largest area of research revolves around improving the electrochemical properties, such as capacitance and energy density. An increase in energy density is of particular importance when trying to reach comparable performances to batteries, since low supercapacitor energy density is one of the biggest current drawbacks. As can be seen in Table 1, novel structures and material combinations are now capable of delivering much higher energy densities than traditional supercapacitors. Many of the biggest recent improvements in supercapacitor performance have been due to the functionalisation of the electrode materials. Depending on the functional group and method chosen, it can lead to improvements in many properties, including specific capacitance, wettability or energy density. Furthermore, the introduction of pseudocapacitive materials, such as metal oxides, onto the carbon surface has had great success in increasing capacitance. Studies have shown the possibility of increasing the capacitance to 4 times that of unmodified materials [51]. Recent work has also aided the scale up of carbon electrode material production, such as the development of ball-milling techniques to produce edge-carboxylated graphene nanoplatelets [53]. However, more progress is still needed in order for these supercapacitors to become commercially viable; both in the production of the carbon materials and the production of the electrodes.

Further important developments that were beyond the scope of this review include the improvement of hybrid supercapacitors, batteries and electrolytes, in particular solid electrolytes. Finally, it must be noted that supercapacitors are unlikely to ever be a full replacement for traditional batteries, and are at their best when used to complement battery storage. Thus, energy storage research must also focus on the wider picture, and how individual technologies can be best utilised and combined to help solve future energy problems.

## Figures and Tables

**Table 1:** Comparative electrochemical performance of various GNP materials.

Material	Structure	Electrolyte	Specific Capacitance F g${}^{-1}$	Current Density/ Scan Rate	Power Density kW kg${}^{-1}$	Energy Density Wh kg${}^{-1}$	Cycle Life	Ref.
GNP	Commercial powder	1 M $TEABF_{4}$/AN ^†^	70	25 mV s${}^{-1}$	5.45	13	-	[51]
GNP	Commercial powder	0.5 M $H_{2}SO_{4}$	242.98	5 mV s${}^{-1}$	-	-	-	[21]
GNP	Commercial powder	0.5 M $Na_{2}SO_{4}$	190.70	5 mV s${}^{-1}$	-	-	-	[21]
GNP	Commercial powder	0.5 M KOH	135.78	5 mV s${}^{-1}$	-	-	-	[21]
GNP	Peanut shell derived	1 M $H_{2}SO_{4}$	186	0.5 A g${}^{-1}$	37.5	58.13	87% (5000 cycles at 10 A g${}^{-1}$)	[54]
GNP	Ball-milled GNP	2 M KCl	100–150 125	0.01–0.2 V s${}^{-1}$ 1 A g${}^{-1}$	-	13.9–20.8	92% (100 cycles at 400 mV s${}^{-1}$)	[52]
GNP	Scalable exfoliated GNP	1 M $TEABF_{4}$/AN ^†^	26	6 A g${}^{-1}$	12.65 at 14 A g${}^{-1}$	12.2 at 6 A g${}^{-1}$	90% (5000 cycles at 14 A g${}^{-1}$)	[57]
GNP	Scalable exfoliated GNP	1 M $Na_{2}SO_{4}$	86	2 A g${}^{-1}$	7.5 at 15 A g${}^{-1}$	11 at 2 A g${}^{-1}$	95% (5000 cycles at 12 A g${}^{-1}$)	[57]
GNP	Flexible GNP electrode	$PVA/H_{3}PO_{4}$ (gel)	380	20 mV s${}^{-1}$	-	79	-	[67]
FLG	Novel combustion synthesised FLG	EMI [TFSI]	222	1 A g${}^{-1}$	35	76.3	99% (8000 cycles at 10 A g${}^{-1}$)	[58]
GNR	Graphene Nanoribbon	1 M $H_{2}SO_{4}$	198 168	50 mA g${}^{-1}$ 1 A g${}^{-1}$	-	-	-	[55]
C-FLG	Microporous carbon/ FLG heterostructure	2 M KOH	128–187	5 mV s${}^{-1}$	-	-	-	[64]
$MnO_{2}$-GNP	$MnO_{2}$-functionalised ball-milled GNP	2 M KCl	450–542 511	0.01–0.2 V s${}^{-1}$ 1 A g${}^{-1}$	-	62.5–75.3	85% (100 cycles at 400 mV s${}^{-1}$)	[52]
GNP-COOH	Edge-carboxylated GNP	1 M $H_{2}SO_{4}$	365.72 (3 electrode) 284.12 (2 electrode)	1 A g${}^{-1}$ 1 A g${}^{-1}$	- 0.22	- 25.25	93% (10,000 cycles at 2 A g${}^{-1}$)	[53]
HRFLG	Hydroxyl-rich FLG	2 M $H_{2}SO_{4}$	320	1 A g${}^{-1}$	6.1	15.4	121% (25,000 cycles at 100 mV s${}^{-1}$)	[66]
LSMCO-GNP	LSMCO perovskite ^‡^/ GNP composite	1 M $H_{2}SO_{4}$	50.11	5 mV s${}^{-1}$	-	4.74–4.20	-	[65]

^†^ TEABF4/AN = tetraethylammonium tetraﬂuoroborate in acetonitrile; ^‡^ LSMCO = La_0.8_Sr_0.2_Mn_0.5_Co_0.5_O_3−δ_.

## Data Availability

Not applicable.

## Abbreviations

The following abbreviations are used in this manuscript:

AC	Activated carbon
BET	Brunauer-Emmett-Teller
CDC	Carbide-derived carbons
CNT	Carbon nanotube
CV	Cyclic voltammetry
EDL	Electric double-layer
EDLC	Electric double-layer capacitor
FLG	Few layer graphene
GCD	Galvanostatic charge-discharge
GNP	Graphene nanoplatelets
LIB	Lithium-ion battery
SSA	Specific surface area
